# CellExcite: an efficient simulation environment for excitable cells

**DOI:** 10.1186/1471-2105-9-S2-S3

**Published:** 2008-03-26

**Authors:** Ezio Bartocci, Flavio Corradini, Emilia Entcheva, Radu Grosu, Scott A Smolka

**Affiliations:** 1 Dipartimento di Matematica e Informatica, Università di Camerino, Via Madonna delle Carceri n.9, Camerino, Italy; 2 Department of Biomedical Engineering, Stony Brook University, NY 11794-4400, USA; 3 Department of Computer Science, Stony Brook University, NY 11794-4400, USA

## Abstract

**Background:**

Brain, heart and skeletal muscle share similar properties of excitable tissue, featuring both discrete behavior (all-or-nothing response to electrical activation) and continuous behavior (recovery to rest follows a temporal path, determined by multiple competing ion flows). Classical mathematical models of excitable cells involve complex systems of nonlinear differential equations. Such models not only impair formal analysis but also impose high computational demands on simulations, especially in large-scale 2-D and 3-D cell networks. In this paper, we show that by choosing Hybrid Automata as the modeling formalism, it is possible to construct a more abstract model of excitable cells that preserves the properties of interest while reducing the computational effort, thereby admitting the possibility of formal analysis and efficient simulation.

**Results:**

We have developed CellExcite, a sophisticated simulation environment for excitable-cell networks. CellExcite allows the user to sketch a tissue of excitable cells, plan the stimuli to be applied during simulation, and customize the diffusion model. CellExcite adopts Hybrid Automata (HA) as the computational model in order to efficiently capture both discrete and continuous excitable-cell behavior.

**Conclusions:**

The CellExcite simulation framework for multicellular HA arrays exhibits significantly improved computational efficiency in large-scale simulations, thus opening the possibility for formal analysis based on HA theory. A demo of CellExcite is available at .

## Background

An excitable cell has the ability to propagate an electrical signal—known at the cellular level as the *Action Potential* (AP)—to surrounding cells. An AP corresponds to a change of potential across the cell membrane, and is caused by the flow of ions between the inside and outside of the cell. The major ion species involved in this process are sodium, potassium and calcium; they flow through multiple voltage-gated ion channels as pore-forming proteins in the cell membrane. Excitation disturbances can occur in the behavior of these ion channels at the cell level, or in the propagation of the electrical waves at the cell-network level. Examples of excitable cells are neurons, cardiac myocytes and skeletal muscle cells. Excitable cell networks are important in the normal functioning and in the pathophysiology of many biological processes. In particular, the rhythmic, pump-like function of the heart is driven by muscle contractions, which are in turn triggered by cell-generated electrical signals (excitations). Of special interest are cardiac arrhythmias: disruptions of the normal excitation process due to faulty processes at the cellular level, single ion-channel level, or at the level of cell-to-cell communication. The clinical manifestation is a rhythm with altered frequency (tachycardia or bradycardia) or the appearance of multiple frequencies (polymorphic Ventricular Tachycardia) with subsequent deterioration to a chaotic signal (Ventricular Fibrillation). VF [1] is a typically fatal condition in which there is uncoordinated contraction of the cardiac muscle of the ventricles in the heart. As a result, the heart fails to adequately pump the blood, and hypoxia may occur.

Excitable tissue is modeled in terms of reaction-diffusion systems. Thus, a typical continuous representation would involve partial differential equations (PDEs) for the diffusing species (typically the transmembrane potential), and a system of nonlinear ordinary differential equations describing all other state variable that are normally considered non-diffusing. These may include ion-channel gating variables and ion concentrations. The first mathematical model of ionic processes that underly cell excitation was empirically developed in 1952 by Hodgkin and Huxley (HH) for a squid giant axon [2]. This provided the basis for subsequent models of increasing complexity, using multiple continuous state variables (voltage, ion-channel gates, ion concentrations) to describe APs in different cell types [3-5]. Current models of cardiac cells include more than 20 such state variables and a large number of fitted parameters. Detailed models of cardiac excitation are perceived as over-determined systems and, as such, make both qualitative—i.e. checking general properties—and quantitative analysis—i.e. by simulation—at the organ or even tissue level impractical. At the opposite end of the spectrum, completely discrete models based on cellular automata (CA) have emerged [6,7].

The first generation of CA models used nearest-neighbor diffusion modeling (Neumann and Moore neighborhoods) and a small number of discrete states, resulting in unrealistic AP shape and wave propagation. Second-generation CA models [7] focused on correct representation of wavefront curvature effects by employing more complex neighborhood functions, such as Gaussian, circular templates or randomized lattices. Furthermore, the transitions rules for the relaxation states were updated to reflect a higher threshold for excitation and to effectively represent the relative and absolute refractory period. The latest generation is exemplified by Barkley's model [8], in which a standard finite-difference method is used to calculate the diffusive term, but CA-like rules govern the kinetics of the two model variables, with adjustable thresholds. Recently, modified CA models have been used to study cardiac excitability and for comparison with experimental data [6,9]. A body of literature provides clear links between the classical continuous PDE representation and the more ad hoc CA-based approach as an alternative description of reaction-diffusion systems. The purely discrete nature of CA presents some difficulties in capturing subtle non-stepwise features of excitation.

One way to reduce the complexity of models based on differential equations while preserving the fundamental features of these systems is to construct a more abstract model that preserves the properties of interest. One promising approach is based on the use of *Hybrid Automata* (HA) [10,11] as a modeling formalism for complex biological processes. More formally, HA are an extension of finite automata that allows one to associate a continuous behavior with each state. The approach of [12] demonstrated the feasibility of using HA as a modeling formalism for excitable cells. The biological behavior of such cells is intrinsically hybrid in nature, featuring both discrete (all-or-nothing response to electrical behavior) and continuous behavior (recovery to rest follows a temporal path, determined by multiple competing ion flows). Starting from a biological interpretation of their APs, 4-state HA models have been derived in [12] for several classical excitable-cell types. In this paper, we present CellExcite, a sophisticated simulation environment for excitable-cell networks based on these 4-state models.

## Results and discussion

**Figure 1:**
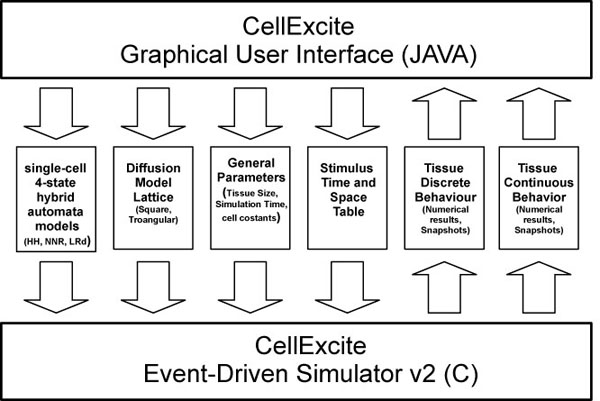
**General Architecture of CellExcite**.

CellExcite allows the user to sketch a tissue of excitable cells, plan the stimuli to be applied during simulation, and customize the diffusion model. As Figure 1 illustrates, the architecture of CellExcite consists of two main components:

**CellExcite Graphical User Interface (GUI)** This component provides several panels that the user can access in order to customize the features of an excitable-cell network. Using the *Tissue Panel*, the user can specify the tissue size, simulation time, and list of stimuli to be applied during simulation. The *Parameters Panel* allows the user to select the desired 4-state hybrid automaton representing the behavior of a particular type of excitable cell: HH for neuron, NNR for cardiac myocytes. Furthermore a user can specify the distance between two neighboring cells, the membrane capacitance, and the time step of the simulation. The *Diffusion Panel* enables the user to select from among different lattices that could better approximate the disposition of cells in a 2D tissue. Through this panel, the user can also choose the radius of the voltage influence a cell has on its neighbors.

**Event-Driven HA-based Simulator** This component has been implemented by extending the event-driven approach described in [13] with the following new features:

• The event priority queue is optimized: duplicate events, i.e. multiple events of the same type for the same cell and time step generated by neighboring cells, are eliminated.

• Several neighborhood functions are added: cell networks can be represented both as a triangular and square lattice. The diffusion and electrical propagation within a cell network are modeled with an exponential neighborhood function.

• Colored snapshots and video representing both the discrete and continuous behavior of the system can be generated.

### An example in-silico simulation of excitable cells

In this section, we provide an example simulation of an excitable-cell network using CellExcite. The goal is to first simulate ventricular fibrillation and then defibrillation on a tissue of NNR (neonatal rat) cardiac myocytes arranged in a 400x400 array. We wish to simulate this network for the first 500 ms with a time step of 1^−3^ ms.

### Sketching a tissue and planning the stimuli to be applied using the Tissue Panel

To carry out the simulation, we must first perform the following operations:

• Resize the tissue to 400x400 cells.

• Set the simulation time to 500 *ms*.

• Apply at the beginning of the simulation, for 1 *ms*, a first stimulus of 800 μ*A/cm*^2^ to a rectangular area of the tissue, from row 310 on the top of the tissue to row 395 on the bottom, and from column 5 on the left to column 15 on the right.

• Apply 145 *ms* after the beginning of the simulation, for 1 *ms*, a second stimulus of 1000 μ*A/cm*^2^ to a rectangular area of the tissue, from row 235 on the top of the tissue to row 245 on the bottom, and from column 0 on the left to column 150 on the right.

• Apply 400 *ms* after the beginning of the simulation, for 1 *ms*, a third stimulus of 800 μ*A/cm*^2^ to a rectangular area of the tissue, from row 4 on the top of the tissue to row 394 on the bottom, and from column 4 on the left to column 394 on the right.

**Figure 2:**
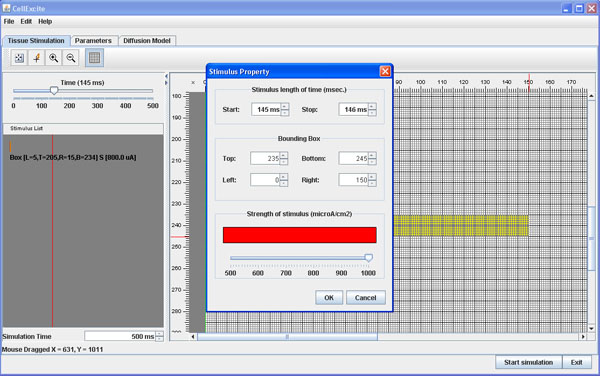
**Planning a stimulus using Tissue Panel**.

As Figure 2 shows, the Tissue Panel of the CellExcite GUI allows the user to insert all these data using a convenient visual framework. Figure 2 contains a snapshot of the GUI while the user is planning the second stimulus on the tissue.

### Setting the cell parameters

The next step is to set the single-cell parameters, such as the choice of 4-state HA model, the distance between two neighboring cells, the cell's membrane capacitance, and the time step of the simulation. For our example, we proceed as follows:

• Choose NNR as the 4-state HA model representing the behavior of a single cell

• Set the distance between two neighboring cells to 0.01 *cm*

• Set the membrane capacitance to 1 μ*F/cm*

• Set the simulation time step to 0.001 *msec*

**Figure 3:**
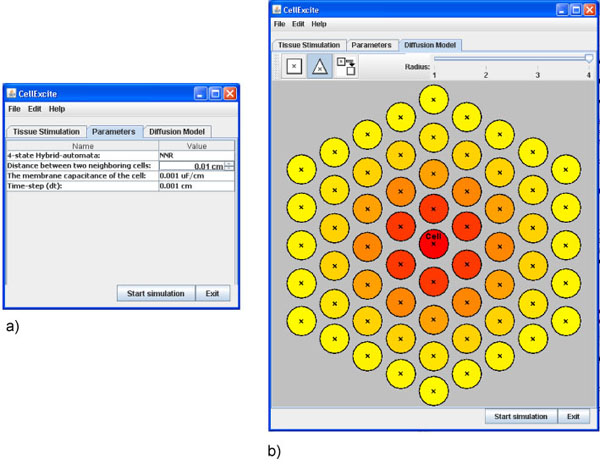
**Setting parameters and diffusion panel**.

Figure 3 a) is a snapshot of the GUI while the user is setting the single-cell parameters.

### Selecting a diffusion model

To complete the experiment, we need to specify the lattice on which we would like to dispose the cell network. The *DiffusionPanel* of CellExcite provides two different lattices: triangular and square. Furthermore, a user can specify a cell's radius of influence with respect to neighbor cells. A gradient of colors, from yellow to red, indicate respectively the minor or major weight, based on a normalized exponential function, of neighbor cells with respect to the cell disposed in the middle of the panel. The higher the weight, the larger the number of neighbor cells taken into account. In this case, the simulation of the diffusion process is more precise but also more computationally intensive, as the number of computations is increased. As Figure 3 b) shows, for our experiment, we choose a triangular lattice with a radius of 4.

### Results of simulation

**Figure 4:**
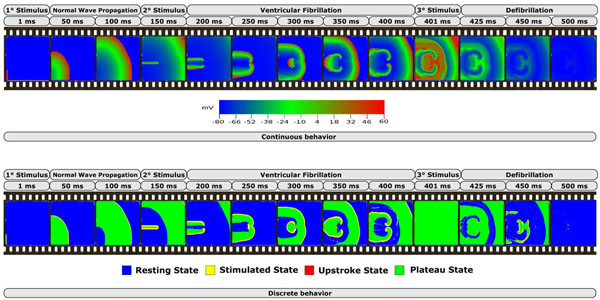
**Results of the simulation**.

Figure 4 shows the results obtained with CellExcite, by simulating our in-silico experiment. The top of the figure depicts the continuous behavior of the tissue during the simulation. This is a quantitative analysis showing the voltage of each cell of the tissue at a particular time step. The bottom of the figure depicts the discrete behavior of the tissue. This is a qualitative analysis showing, for each cell of the tissue and for each time step, the AP state of the hybrid automaton: resting, stimulated, upstroke and plateau. As the sequence of pictures shows, the first stimulus has a normal propagation, while the second generates two counter-clockwise spirals because there is not enough space for normal propagation. After 150 *ms*, the heads of the two spirals collide and generate other spirals. This degeneration of the electrical propagation is the cause of fibrillation. A third stimulus at is applied at 400 *ms* to all cells in the tissue in order to recover them the resting state. This phenomena is comparable to defibrillation.

### Performance comparison

To illustrate the performance gains achieved in an HA-based simulation framework for excitable cells versus a traditional ODE-based one, consider Table 1. There we give the CPU times required for the two frameworks on a 2-second simulation of NNR tissue, with the tissue size ranging from a 2x2 cell array to a 400x400 cell array. On the more substantial cases (i.e., array sizes of 16x16 on up), it can be seen that the HA-based simulation framework enjoys an almost 8-fold speedup over the ODE-based one. All results, which were originally reported in [12], were produced on a PC equipped with a Pentium Intel 4 CPU 3.00GHz with 1GB of memory.

**Table 1:** Performance comparison for 2-second simulation

cell array size	original	hybrid
2 × 2 cell array	5 s	3 s
4 × 4 cell array	9 s	3 s
8 × 8 cell array	26 s	6 s
16 × 16 cell array	93 s	14 s
32 × 32 cell array	365 s	51 s
64 × 64 cell array	1460 s	198 s
400 × 400 cell array	17 h 10 m 33 s	2 h 13 m 38 s

### Availability and requirements

• **Project Name:** Excitable Hybrid Automata (EHA)

• **Project HomePage:**[14]

• **Operating System:** Windows, Linux

• **Programming Language:** C, Java

• **Licence:** The CellExcite software package is available under the GNU Less General Public License (LGPL) license. Please contact the first author for details.

## Conclusions and future work

We developed CellExcite, a hybrid-automata-based visualization framework for excitable-cell networks. CellExcite provides a user interface that allows the user to sketch a tissue of excitable cells, plan the stimuli to be applied during simulation, and customize the diffusion model. The use of multicellular HA to model networks of excitable cells is a reasonable compromise to reduce the computational demands of large scale 2D cell-network simulations, without losing the ability to capture important features of the action potential such as restitution and refractoriness.

Besides quantitative analysis obtained by simulation, this formal model opens up the possibility of qualitative analysis. To this end, as future work, we aim to extend CellExcite with tools that allow the run-time verification of specific spatio-temporal patterns such as the creation of dangerous spirals. This could be helpful to find automatically an energy-efficient strategy to counteract degeneration of the normal electrical propagation. This model-based control could be exploited for the construction of the next-generation cardio defibrillator. Furthermore, we would like to exploit agent-based technology to better distribute the computation on a grid-computing environment.

## Methods

### Action potential

The electrical signal at the cellular level is known as an *action potential* (AP). Action potentials for ventricular cells (the major cells in the heart) are externally triggered events: a cell fires an action potential as an all-or-nothing response to a supra-threshold electrical signal, and each AP follows more or less the same sequence of events and has the same magnitude regardless of applied stimulus. An AP lasts for a couple of hundred milliseconds in most mammals. During the AP, no re-excitation can occur, which is a safety mechanism to ensure the reliable working of the heart. The early portion of an AP is known as the “absolute refractory period” due to its non-responsiveness to further simulation. The later portion of an AP is known as the “relative refractory period”, during which an altered secondary excitation event is possible if the stimulation threshold is raised.

Despite differences in AP duration, morphology and underlying ion currents between different species and different regions in the heart, the following major AP phases can be identified: resting phase, rapid upstroke, early repolarization phase, plateau or later repolarization phase, and final repolarization (identical to the resting state due to the cyclic nature of an AP). The resting state features a constant transmembrane potential (difference between the inside and outside potential of the cell) of about −80 mV for most species; i.e. the membrane is polarized at rest. During the AP upstroke, the transmembrane potential rapidly changes (over the course of a couple of milliseconds) from negative to positive; i.e. the membrane depolarizes. This is followed by an early repolarization phase. A slower, plateau phase is present in most mammalian action potentials, during which calcium influx facilitates the muscle contraction. After this phase, a faster initial repolarization brings the potential back to the resting state. Because of the universal nature of these AP features between species and regions, we use them as a guide in the construction of HA models [12].

### The Hodgkin-Huxley Model

The first quantitative description of cellular excitation was empirically developed by Hodgkin and Huxley (HH) for a squid giant axon [2]. The HH model includes three ionic currents: fast inward sodium, outward potassium, and a time-independent linear (leak) current. The generalized form of HH model is as follows:

$$\begin{array}{c} C\dot{V}\;=\;-{\bar{g}}_{N_{a}}m^{3}h\left(V\;-\;E_{Na}\right)\;-\;{\bar{g}}_{K}n^{4}h\left(V\;-\;E_{K}\right)\;-\;{\bar{g}}_{L}\left(V\;-\;E_{L}\right)\;+\;I_{st}\\ \dot{y}=\left(y-y_{\inf}\right)/\tau ,y_{\inf}=\varphi_{y}\left(V\right),y\leftarrow m,h,n \end{array}$$

where V is the transmembrane voltage [mV], whose variation forms the AP; ${\bar{g}}_{Na},\;\;{\bar{g}}_{K}\;,\;{\bar{g}}_{L}$ are the maximum channel conductance [mS/μ F] for the sodium (Na), potassium (K) and the leakage channel (L), respectively; *E_Na_*, *E_K_*, *E_L_* are the reversal potentials [mV] for the sodium, potassium and the leakage channel, respectively; *m*, *h*, *n* are voltage- and time-dependent ion channel gates, following the same general differential equation in *y*, where *y*_inf_ and τ_inf_ represent the steady-state and the time constant of a gate; C is the cell capacitance [μ*F*], and *I_st_* is the stimulation current [μ*A*/μ*F*].

### Luo-Rudy Guinea Pig Ventricular Cell Model

In a series of papers, Y Rudy et al. have developed some of the most detailed cardiac cell models to date, targeting guinea pig [5,15]. The ion-channel description in these models follows the same framework as the HH model, but a much larger number of ion currents is included. The complexity of this class of models is further increased by the addition of active ion pumps, intracellular compartments for calcium transport and calcium buffers. The detailed description of the LRd model is omitted here.

### Neonatal Rat Ventricular Cell Model

Among the mammalian species, the mouse and the rat have a substantially different AP morphology—much more triangular with almost absent plateau phase—compared to the AP simulated by the LRd model. Neonatal rats are often used as an experimental model in cardiac electrophysiology, and a computational model is a derable tool. A neonatal rat model (NNR) is being developed by Entcheva et al., derived from the LRd model. In [12], Pye et al. use a hybrid-automaton formulation (following the same structure as for LRd) with adjusted parameters to replicate the behavior of this detailed ionic model.

### Modeling Action Potential using Hybrid Automata

To permit formal analysis and to increase simulation efficiency, it could be useful to perform abstraction on a set of nonlinear differential equations describing the behavior an excitable cell to obtain a hybrid automaton. Formally, a hybrid automaton is defined as follows.

A **Hybrid automaton H** consists of the following [11]:

- As finite set *X* = *x*_1_, ⋯, *x_n_* of real-numbered *variables*. The number *n* is called the dimension of H. We write $\dot{X}\;=\;{\dot{x}}_{1},\;\cdots ,\;{\dot{x}}_{n}$ of dotted variables (which represent first derivatives of variables in X), and *X*′ = x′_1_,⋯, *x*′*_n_* of primed variables (which represent values of variables X at the conclusion of discrete steps).

- A finite, discrete control graph (V,E). The vertices in *V* are called control modes. The edges in E are called control switches.

- Three vertex labeling functions init, inv and flow that assign to each control mode e υ ∈ *V* three predicates. Each initial condition init(v) and invariant condition inv(v) is a predicate whose free variables are from X. Each flow condition flow(v) is a predicate whose free variables are from $X\cup \dot{X}$.

- An edge labeling function jump that assigns to each control switch *e* ∈ *E* a predicate. Each jump condition jump(e) is a predicate whose free variables are from *X* ∪ *X*′

- A finite set Σ of events, and an edge labeling function event that assigns to each control switch an event.

The HA models chosen have four control modes: *resting* and *final repolarization* (FR), *stimulated*, *upstroke* and, *plateau and early repolarization* (ER). Initially, the cell is in the resting and final repolarization mode. When (externally) stimulated with the event *V_S_*, the cell enters the stimulated mode and updates its voltage according to the stimulus current. Upon termination of the stimulation, via event ${\bar{V}}_{S}$, with a sub-threshold voltage, the cell returns back to resting mode without firing AP. If the stimulus is supra-threshold, i.e., *V_s_* >*V_T_* holds, the excited cell will generate an action potential by progressing to the upstroke mode. The recovery course of the cell follows the transitions to mode plateau and early repolarization and then to resting and final repolarization. The guards on the control switches monitor the transmembrane potential, rather than imposing a rigid timing scheme. This approach allows for AP adaptation (response to various pacing frequencies).

### HA for the HH model

**Figure 5:**
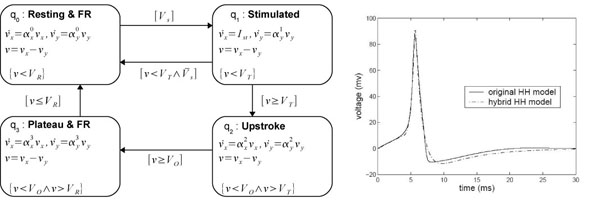
**Hybrid automata for HH model**.

The HA for the HH model is shown in Figure 5. Variables υ_*x*_ and υ_*y*_ define a second-order system of linear differential equations in each control mode. They are an abstraction of the ionic currents and the gates. *I_st_* is the excitation current and *V_S_* is the stimulation event. The membrane voltage is V = υ_x_ − υ_y_. The initial control mode is *q_0_*. The mode invariants are given below the differential equations describing the transmembrane voltage. Like the switch guards, they depend on three model-specific (see Table 2): *threshold voltage V_T_*, *overshoot voltage V_O_*, and *repolarization voltage V_R_*. The transition guards are bracketed.

### HA for the LRd model

The modeling framework for the LRd model is similar to that for HH. However, to properly represent the longer maintained plateau phase of the cardiac AP and to capture its frequency adaptation, we extend the hybrid model with additional variables. A third continuous variable, υ_*z*_, is added. The need for such a variable in the LRd and NNR models can be explained by the major difference in the ion fluxes between neurons and cardiac cells; namely, calcium flux plays a profound role in the maintenance of the AP plateau for proper cardiac muscle contraction to take place.

**Figure 6:**
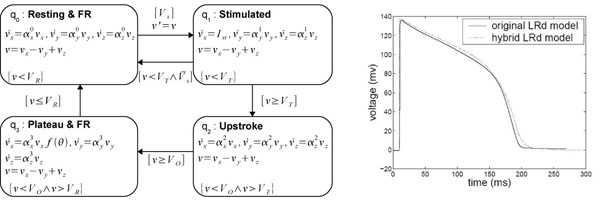
**Hybrid automata for LRd model**.

Additionally, a new restitution-related continuous variable υ′ is added to the LRd model, which is used to modify the overall voltage by reflecting changes in the diastolic interval (DI). DI is the time period between AP's recovery and a new stimulation, i.e., the rest period between successive stimulations. It is known that the immediate memory of an excitable cell is directly linked to the DI: a shorter DI results in a shorter following AP, while a longer DI produces a longer AP [16]. This simple memory model helps capture the proper response of AP to pacing frequency, which is an essential feature of the cardiac excitation. The resulting system of differential equations in the corresponding modes is however no longer linear. The hybrid automaton used to simulate LRd model is defined in Figure 6 with $f\left(\theta \right)=1+13\sqrt{\theta 6}$.

### HA for the NNR model

**Figure 7:**
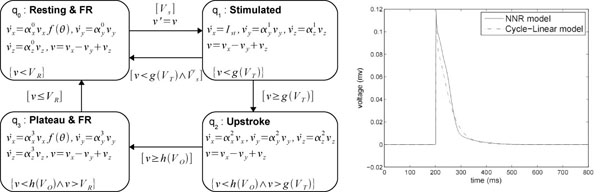
**Hybrid automata for NNR model**.

The HA for NNR is defined in Figure 7. Here, *f*(θ) = 1 + 2θ. For improved modeling of cell-cell interactions in cardiac excitation, the threshold voltages do not remain constant during simulation; instead, they also become a function of θ = *V*′/*V_R_*, defined as follows: $g\left(V_{T}\right)=V_{T}\cdot \left(1+1.45\;\sqrt{\theta 6}\right)$ and $h\left(V_{o}\right)=V_{o}-40\cdot \sqrt{\theta }$

### Parameters definitions

The values of the coefficients and constants occurring in the HH, LRd and NNR HA models are summarized in Table 2. They were obtained either from the cited literature or empirically through experimentation.

**Table 2:** Parameters for HH LRd and NNR 4-state HA models

	*V_R_*	*V_T_*	*V_O_*	*α^0^_x_*	*α^0^_y_*	*α^0^_z_*	*α^1^_x_*	*α^1^_y_*	*α^1^_z_*	*α^2^_x_*	*α^2^_y_*	*α^2^_z_*	*α^3^_x_*	*α^3^_y_*	*α^0^_z_*
HH	10	10	83	−0.98	−0.16	N/A	N/A	−0.16	N/A	1.4	15	N/A	−0.98	−0.16	N/A
LRd	20	20	138	−0.1	−0.1	−0.1	N/A	−0.1	−0.1	200	0	100	−0.001	0.036	0.008
NNR	20	30	120	−0.025	−0.07	−0.2	N/A	−0.07	−0.2	250	200	125	−0.025	−0.07	−0.2

### Diffusion Modeling in Multicellular HA

**Figure 8:**
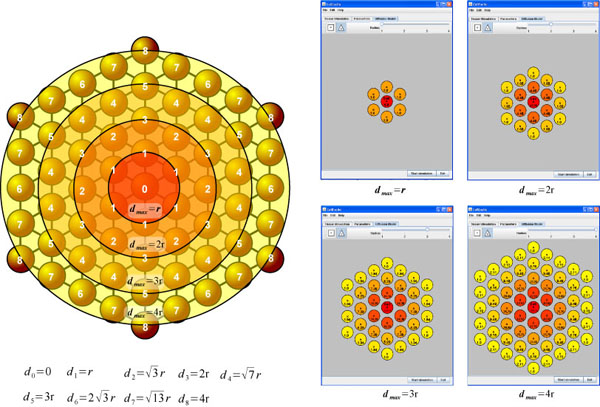
**Triangular lattice**.

**Figure 9:**
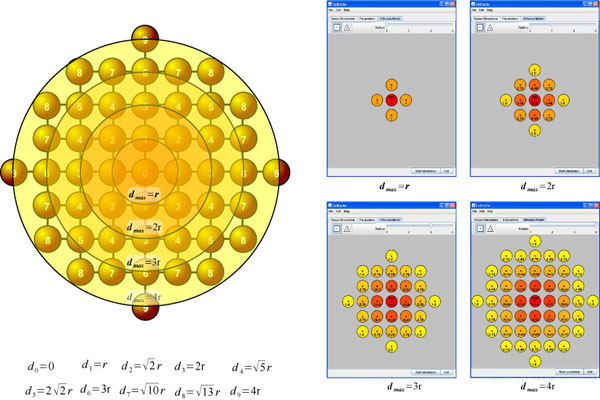
**Square lattice**.

The spatially extended model of multiple connected excitable cells is defined as below, where the left-hand side represents the diffusion term (propagation of the transmembrane voltage), while the right-hand side describes the activity at each cell, according to the selected model (detailed, PDE-based (1) or hybrid (2)):

(1)

$$\nabla \left(\sigma \cdot V\right)=\beta \left(C\delta V/\delta t+I_{ion}-I_{st}\right),$$

(2)

$$\nabla \left(\sigma \cdot V\right)=\beta \left(C\delta V/\delta t+\delta HA/\delta t-I_{st}\right)$$

where ∇ is the Laplacian Operator in 2D; σ is the 2x2 conductivity tensor; β [*cm*^−1^] is the surface area to volume ratio for the cells; *I_ion_*[*mA*/*cm*^2^] is the sum of all ion currents and *I_st_* is the stimulus current. In a preliminary study we coupled the HA description of each cell to classical Laplacian-based diffusion modeling equations, solved by a finite element numerical scheme [13]. Due to the matrix operations involved, this solution is not the most computationally efficient. In this study we extended the spatial modeling of coupled hybrid automata cells by employing alternative diffusion modeling operators. For correct modeling of the diffusion of the electrical potential, simulation data have to be consistent with the above equation for the medium of interest. The Laplacian-based solution of the diffusion term has been substituted with optimized neighborhood functions to match the experimentally obtained dispersion curve, since the latter requires extended neighborhoods, thus slowing computations. The exponential neighborhood function that we used is defined as follows:

$$\nu_{r_{max}}\left(d\right)=e^{\frac{-d^{2}}{r_{max}}+r_{max}}$$

where *r_max_* is maximum radius of influence of a cell respect to its neighbor cells and *d* <*r_max_* is the distance of a cell from a particular neighbor cell. This function provides the weight that assume the voltage of each cell respect to the others. The closer a cell is to its neighbors, the greater the influence of its voltage on its neighbor cells' voltage in the next time step. This function is dependent on the chosen lattice topology. As Figures 8 and 9 illustrate, in CellExcite both triangular and square lattices are supported. Table 3 and 4 report the weights of the cells for each lattice as a function of the distance.

**Table 3:** Weights calculated using neighborhood function for triangular lattice

#	**0**	**1**	**2**	**3**	**4**	**5**	**6**	**7**	**8**
**d**	0	*r*	$\sqrt{3}r$	2*r*	$\sqrt{7}r$	3*r*	$2\sqrt{3}r$	$\sqrt{13}r$	4*r*
$\overset{\Delta }{\nu_{r}}\left(d\right)$	−6	1	*N/A*	*N/A*	*N/A*	*N/A*	*N/A*	*N/A*	*N/A*
${\overset{\Delta }{\nu }}_{2r}\left(d\right)$	−42.96	4.48	1.68	1	*N/A*	*N/A*	*N/A*	*N/A*	*N/A*
${\overset{\Delta }{\nu }}_{3r}\;\left(d\right)$	−191.70	14.39	7.38	5.29	1.94	1	*N/A*	*N/A*	*N/A*
${\overset{\Delta }{\nu }}_{2r}\left(d\right)$	−726.24	42.52	25.79	20.08	9.48	5.75	2.72	2.12	1

**Table 4:** Weights calculated using neighborhood function for square lattice

#	**0**	**1**	**2**	**3**	**4**	**5**	**6**	**7**	**8**	**9**
**d**	0	*r*	$\sqrt{2}r$	2*r*	$\sqrt{5}r$	$2\sqrt{2}r$	3*r*	$\sqrt{10}r$	$\sqrt{13}r$	4*r*
${\overset{□}{\nu }}_{r}\left(d\right)$	−4	1	*N/A*	*N/A*	*N/A*	*N/A*	*N/A*	*N/A*	*N/A*	*N/A*
${\overset{□}{\nu }}_{2r}\left(d\right)$	−32.8	4:48	2.72	1	*N/A*	*N/A*	*N/A*	*N/A*	*N/A*	*N/A*
${\overset{□}{\nu }}_{3r}\left(d\right)$	−159.88	14:39	10.31	5.29	3.79	1.40	1	*N/A*	*N/A*	*N/A*
${\overset{□}{\nu }}_{4r}\left(d\right)$	−636.52	42:52	33.12	20.08	15.64	12.18	5.75	4.48	2.12	1

## List of abbreviations used

• AP: Action Potential

• CA: Cellular Automata

• DI: Diastolic Interval

• GUI: Graphical User Interface

• HA: Hybrid Automata

• HH: Hodgkin Huxley

• LGPL: Less General Public License

• NNR: Neo-Natal Rat

• PDE: Partial Differential Equation

• VF: Ventricular Fibrillation

## Competing interests

The authors declare that they have no competing interests.

## Authors' contributions

EB wrote the paper and participated in the tool design and development. FC, EE, RG and SAS conceived the study, coordinated the tool design, and helped to draft the manuscript. All authors read and approved the final manuscript.
